# Comparative analysis of ultrasound-guided magnetic resonance imaging-cognitive fusion transrectal versus transperineal prostate biopsy: a 10-year single-center retrospective analysis

**DOI:** 10.3389/fmed.2025.1713863

**Published:** 2025-11-19

**Authors:** Jincheng Luo, Jie Sun, Hui Wang, Cheng Yang

**Affiliations:** 1 Department of Urology, The First Affiliated Hospital of Anhui Medical University, Institute of Urology, Anhui Medical University, Hefei, China; 2 Anhui Province Key Laboratory of Urological and Andrological Diseases Research and Medical Transformation, Anhui Medical University, Hefei, China; 3 Department of Urology, The Fourth People’s Hospital of Lu’an, Lu’an, China

**Keywords:** prostate cancer, ultrasound-guided, transperineal prostate biopsy, magnetic resonance imaging-cognitive fusion, complications

## Abstract

**Objectives:**

Prostate cancer (PCa) remains a leading malignancy among men worldwide. The diagnostic approach, particularly the biopsy route and integration with imaging, is crucial for accuracy. This study aimed to directly compare the diagnostic efficacy and safety of two ultrasound-guided, MRI-cognitively fused prostate biopsy approaches using a consistent extended 12 + X-core sampling scheme: the transperineal (TPB) versus the transrectal (TRB) route.

**Methods:**

We conducted a comparative, retrospective analysis of 3,208 patients who underwent prostate biopsy at our institution between January 2015 and January 2024. Patients were categorized into two cohorts: a historical TRB cohort (*n* = 1,078) from 2015–2018 and a subsequent TPB cohort (*n* = 2,130) from 2018–2024. Crucially, both cohorts were investigated using an identical 12 + X-core protocol under MRI-cognitive fusion guidance. Pathological outcomes, PCa detection rates, and perioperative complications were systematically compared. Multivariable logistic regression was employed to identify predictors of PCa detection.

**Results:**

The TPB cohort demonstrated a significantly higher overall PCa detection rate (55.73% [1,187/2,130]) compared to the TRB cohort [50.46% (544/1,078); ^*^p < 0.05]. Furthermore, TPB was associated with a superior safety profile, with minor complications (e.g., hematuria, low-grade fever, transient urinary symptoms) occurring in only 5.82% (124/2,130) of cases. Multivariable analysis confirmed established clinical predictors for PCa. Stratification of the detected cancers revealed that 1,701 patients (63.85%) were diagnosed with high-risk disease (Gleason score ≥8), outlining the distribution within our PCa population.

**Conclusion:**

In this comparative study, the ultrasound-guided TPB with MRI-cognitive fusion and a 12 + X-core protocol demonstrated superior diagnostic efficacy and a more favorable safety profile compared to the TRB. These findings support the adoption of the TPB approach as a preferred clinical strategy for prostate biopsy.

## 1 Introduction

Prostate cancer (PCa) remains one of the most prevalent malignancies in men worldwide (1). Surveillance data from the Cancer Monitoring Center indicate a steady annual rise in both its incidence and mortality rates (2). Currently, PCa ranks as the second most common urological malignancy and the sixth leading cause of cancer-related deaths globally (3). Since the 1990s, the diagnostic paradigm for PCa has evolved to incorporate digital rectal examination (DRE), prostate-specific antigen (PSA) testing, multiparametric magnetic resonance imaging (mp-MRI), and biopsy (4), with histopathological biopsy remaining the gold standard for definitive diagnosis (5). The two primary biopsy approaches include transrectal ultrasound (TRUS)-guided transrectal prostate biopsy (TRB) and transperineal prostate biopsy (TPB). While TRB is technically simpler and more widely adopted in clinical practice, it carries a higher risk of undersampling the apical and anterior prostate regions—a limitation effectively addressed by TPB (6). Furthermore, accumulating evidence demonstrates that TPB is associated with a lower incidence of infectious and hemorrhagic complications compared to TRB (7). The integration of mp-MRI has revolutionized prostate biopsy, among which the MRI-ultrasound fusion-guided biopsy technology based on the Prostate Imaging Reporting and Data System (PI-RADS) scores has become a state-of-the-art diagnostic tool (8, 9). This cognitive fusion technique not only improves the detection rate of clinically significant PCa but also mitigates the overdiagnosis of indolent, low-risk disease, thereby gaining increasing clinical adoption for precise diagnosis and personalized treatment planning.

Beyond anatomical imaging, molecular imaging has revolutionized staging. Prostate-specific membrane antigen (PSMA) positron emission tomography/computed tomography (PET/CT) has emerged as a highly sensitive method for detecting metastatic disease. While it demonstrates excellent overall diagnostic accuracy, its limitations in specific high-risk contexts—such as Grade Group 5 (GG5) cancer and ductal adenocarcinoma—underscore the need for complementary prognostic tools and the continued role of surgical lymph node dissection in definitive staging (10). Concurrently, investigation into the tumor microenvironment has identified immune biomarkers such as PD-L1, which are elevated in aggressive diseases like GG5 and cribriform patterns and predict a higher risk of biochemical recurrence, highlighting their potential to guide immunotherapy and improve risk stratification (11). However, economic costs have restricted its wide promotion.

Despite these advances, the optimal biopsy core number remains controversial. Current strategies include: systematic biopsy (e.g., the conventional 12-core protocol), which provides comprehensive prostate sampling to minimize sampling error, and targeted biopsy (typically 2–4 cores per lesion), which focuses on MRI-identified suspicious lesions. To reconcile these approaches, our institution developed a tailored “12 + X-core” protocol, combining the standard 12-core systematic biopsy with additional targeted cores (X) corresponding to the number of MRI-visible lesions (X). This study evaluated the diagnostic advantages of this hybrid strategy between TRB and TPB with MRI-cognitive fusion using 12 + X-core protocol.

## 2 Materials and methods

### 2.1 Study population

**Figure 1:**
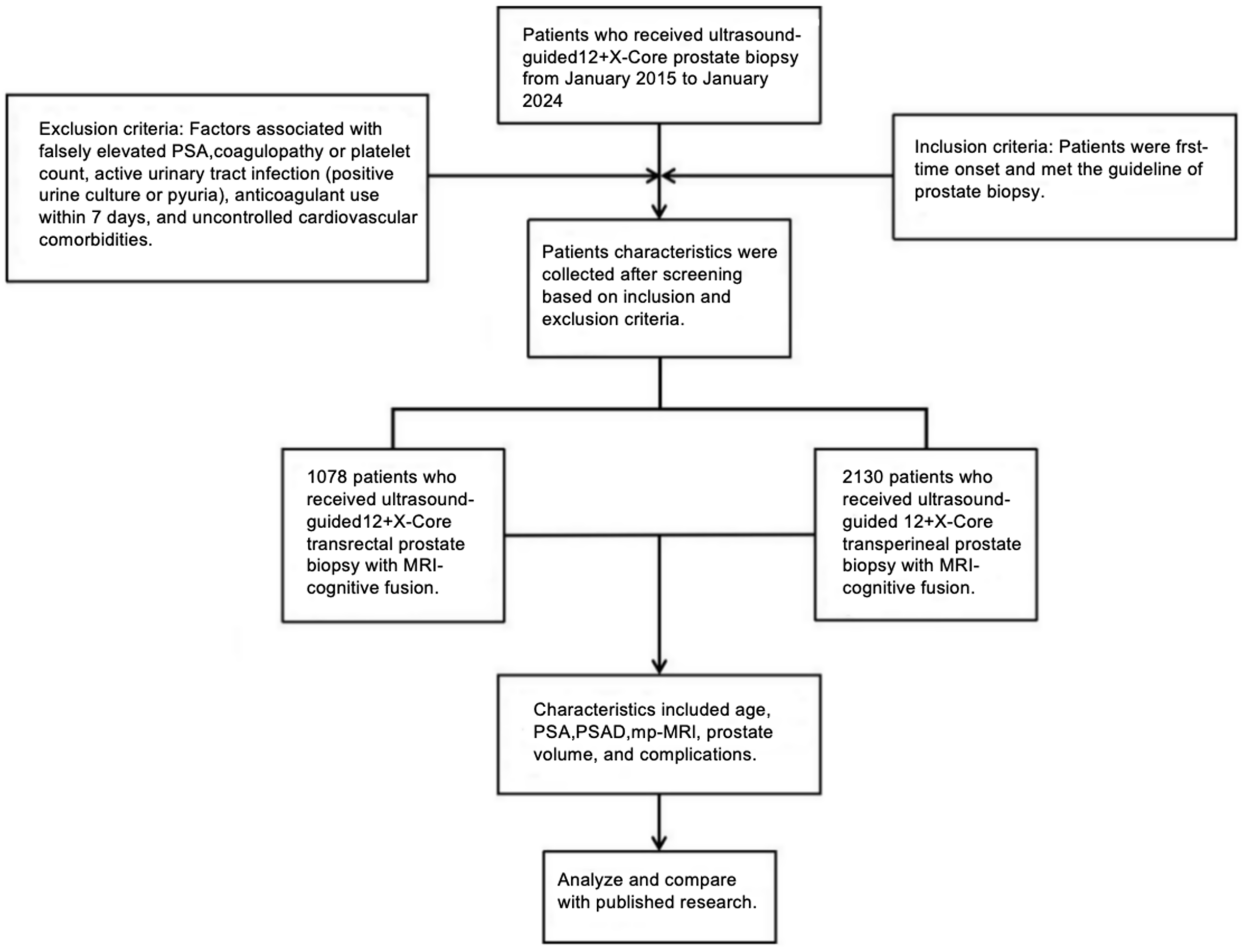
Flowchart of the design process of this study.

Patients exhibiting clinical abnormalities meeting guideline-recommended indications for prostate biopsy underwent transrectal ultrasound (TRUS)-guided biopsy at our institution. We retrospectively analyzed de-identified clinical data from eligible patients recorded in the electronic medical system between January 2015 and January 2024. Two biopsy techniques were employed: (1) MRI-cognitively fused TRUS-guided TRB (12 + X-core scheme) from 2015 to 2018, (2) MRI-cognitively fused TRUS-guided TPB (12 + X-core scheme) from 2018 to 2024. After screening, a final cohort meeting inclusion criteria was established (Figure 1). Inclusion criteria is treatment-naïve patients meeting contemporary guideline criteria for prostate biopsy. Exclusion criteria included factors associated with falsely elevated PSA, coagulopathy (INR >1.5 or platelet count <50,000/μL), active urinary tract infection (positive urine culture or pyuria), anticoagulant use within 7 days, and uncontrolled cardiovascular comorbidities.

### 2.2 Biopsy protocol

Before biopsy, all patients underwent multiparametric MRI. Two experienced radiologists interpreted the images according to PI-RADS criteria and identified suspicious lesions. For the transrectal biopsy (TRB) cohort (2015–2018), MRI cognitive fusion-targeted biopsy was performed as follows: The patient was placed in the left lateral decubitus position. Prior to biopsy, the operators (two fixed urologists from the same team, both with extensive experience) carefully reviewed the mp-MRI images on a workstation to familiarize themselves with the exact location, size, and spatial relationships of suspicious lesions relative to anatomical landmarks. Under real-time TRUS guidance, the operator mentally fused the memorized MRI lesion information with the live ultrasound image to identify the target. An 18-gauge automatic biopsy needle was then used transrectally to obtain 2–3 targeted cores from each suspicious lesion under continuous ultrasound monitoring. All patients also underwent systematic biopsy (typically 12 cores), regardless of MRI findings. For the transperineal biopsy (TPB) cohort (2018–2024), the cognitive fusion principle was identical to that used in the TRB group. Key differences included patient position, anesthesia, and puncture route: patients were placed in the lithotomy position, typically under intravenous or caudal anesthesia. After perineal disinfection, the ultrasound probe was inserted transrectally. Targeted sampling (2–3 cores per lesion) was performed via the transperineal route using a biopsy needle, followed by routine template systematic biopsy (typically 12–16 cores) (12).

### 2.3 Statistical analysis

Continuous variables with normal distribution (assessed by Shapiro–Wilk test) are reported as mean ± standard deviation (SD) and compared via Student’s *t*-test. Non-parametric data used Mann–Whitney *U* test. Categorical variables employed chi-square or Fisher’s exact tests. Multivariate logistic regression (list covariates adjusted for) evaluated independent predictors, reporting adjusted odds ratios (OR) with 95% confidence intervals (CI). Analyses used SPSS 27.0 (IBM Corp.), with two-tailed *p* < 0.05 deemed significant.

## 3 Results

### 3.1 Characteristic of pathological results

The study analyzed 2,130 consecutive TPB procedures. Pathological examination revealed 943 cases of benign prostatic hyperplasia (BPH) and 1,187 cases of PCa, yielding a PCa detection rate of 55.73% (1,187/2,130). In comparison, a historical TRB cohort of 1,078 patients showed 534 BPH cases and 544 PCa cases, with a significantly lower detection rate of 50.46% (544/1,078, *p* < 0.05). We compared PCa and BPH patients across multiple parameters, including age, PSA levels, PSA density (PSAD), PI-RADS scores, and prostate volume (PV) (details in Table 1). Multivariate regression analysis identified these variables as statistically significant independent predictors of PCa (Table 2). Through univariate and multivariate analyses, only these five indicators demonstrated independent statistical significance (*p* < 0.01), as shown in prior findings. Age, PSA, and PSAD are well-established risk factors with clear clinical relevance, while PI-RADS v2—assigned triple the weight of other variables—proved to be the strongest predictor, reflecting the critical role of mp-MRI in PCa detection. PV was included due to its inverse association with cancer risk, consistent with clinical observations. These variables collectively formed the Prostate Biopsy Rating Scale (PBRS), which achieved an AUC of 0.87, outperforming any single predictor. Other candidate variables were excluded as they did not retain statistical significance in the multivariate model, ensuring model parsimony and clinical applicability (13). Notably, 32.76% of BPH patients had PI-RADS scores >3, suggesting that prostatitis and other conditions may compromise the diagnostic specificity of PI-RADS, which consistent with prior TRB studies (14).

**Table 1:** Clinical characteristics and demographics of patients.

Characteristics		TRB				TPB			
		Total	BPH	PCa	*p*	Total	BPH	PCa	*p*
Age (mean ± SD)		68.90 ± 8.28	66.85 ± 8.22	70.91 ± 7.85	<0.01	68.66 ± 8.64	65.96 ± 8.5	70.8 ± 8.14	<0.01
PSA (mean ± SD)		30.95 ± 10.90	17.90 ± 8.31	43.76 ± 15.11	<0.01	30.93 ± 32.19	14.85 ± 13.96	43.71 ± 36.56	<0.01
PSAD (mean ± SD)		0.74 ± 0.90	0.40 ± 0.51	1.07 ± 1.07	<0.01	0.8 ± 1.62	0.28 ± 0.28	1.21 ± 2.07	<0.01
PI-RADS (mean ± SD)		3.85 ± 1.00	3.19 ± 0.81	4.38 ± 0.80	<0.01	3.85 ± 1	3.19 ± 0.81	4.38 ± 0.8	<0.01
PI-PADS *n* (*n*/&, %)	2	224 (20.78)	196 (36.70)	28 (5.15)		228 (10.70)	183 (19.41)	45 (3.79)	
	3	331 (30.71)	208 (38.95)	123 (22.61)		551 (25.87)	451 (47.83)	100 (8.43)	
	4	306 (28.39)	114 (21.35)	192 (35.29)		655 (30.75)	257 (27.25)	398 (33.53)	
	5	217 (20.12)	16 (3.00)	201 (36.95)		696 (32.68)	52 (5.51)	644 (54.25)	
PV (mean ± SD)		53.87 ± 36.22	58.76 ± 36.15	49.08 ± 35.68	<0.01	53.45 ± 37.27	62.49 ± 37.96	46.26 ± 35.11	<0.01
Number (&)		1,078	534	544		2,130	943	1,187	

PSA, prostate-specific antigen; PSAD, PSA density; PV, prostate volume; BPH, benign prostatic hyperplasia; PI-RADS, Prostate Imaging Reporting and Data System; PCa, prostate cancer; TRB, transrectal prostate biopsy; TPB, transperineal prostate biopsy; SD, standard deviation.

**Table 2:** The results of multivariate stepwise logistic regression.

Indicators	*B*	S.E.	Wals value	*p*	OR (95% CI)
Age	0.06	0.01	62.23	<0.01	1.06 (1.05–1.08)
PSA	0.02	0.01	7.95	<0.01	1.02 (1.01–1.04)
PSAD	0.11	0.26	8.18	<0.01	1.14 (0.80–2.13)
PI-RADS	1.26	0.08	262.75	<0.01	3.53 (3.04–4.12)
PV	−0.02	0.00	32.76	<0.01	0.98 (0.97–0.99)

PSA, prostate-specific antigen; PSAD, PSA density; PV, prostate volume; PI-RADS, prostate imaging report and data system.

Statistical analyses revealed strong positive correlations between PCa detection rates and age, serum PSA levels, and PI-RADS scores. The oldest cohort (>80 years) exhibited an 82.78% positivity rate, whereas all patients aged <40 years had benign pathology, underscoring age as a relevant screening factor. At a PSA threshold of 100 μg/L, the positive predictive value reached 99.26%. PI-RADS scores also demonstrated incremental diagnostic accuracy, with PI-RADS 5 lesions showing a 92.53% confirmation rate (Table 3). PCa prevalence varied markedly across age groups, PSA ranges, and PI-RADS categories. To evaluate the synergistic effects of PSA and PI-RADS on detection rates, we stratified patients into three age groups: <50, 50–75, and >75 years old.

**Table 3:** The distribution of patients with BPH and PCa in different levels of PSA, age, and PI-PADS.

Variate	Total	BPH	PCa	Detection rate (%)
Total	2,130	943	1,187	55.73
Age (years)				
<40	6	6	0	0
40–60	370	240	130	35.14
61–70	832	402	430	51.68
71–80	742	264	478	64.42
81–100	180	31	149	82.78
PSA (μg/mL)				
<4	9	7	2	22.22
4–9	602	399	203	33.72
10–19	665	366	299	44.96
20–39	339	133	206	60.77
40–59	119	21	98	82.35
60–79	76	10	66	86.84
80–99	48	5	43	89.58
≥100	272	2	270	99.26
PI-RADS				
1	0	0	0	0
2	228	183	45	19.74
3	551	451	100	18.15
4	655	257	398	60.76
5	696	52	644	92.53

PSA, prostate-specific antigen; BPH, benign prostatic hyperplasia; PCa, prostate cancer; PI-RADS, prostate imaging report and data system.

### 3.2 Sub-analysis of detection rates of PCa in different PSA, PI-RADS and age groups

The results demonstrated that the combination of elevated PSA (>20 ng/mL) and PI-RADS 5 yielded a PCa detection rate of nearly 99% in elderly patients (>75 years), underscoring the strong predictive value of integrating Age, PSA, and PI-RADS scores. In contrast, for patients with low PSA (<10 ng/mL), the PI-RADS 5 group achieved a detection rate of 74.47% in the 50–75 age group, highlighting the complementary role of imaging scores in this population, albeit with limited efficacy. Notably, PI-RADS 4 lesions showed significant diagnostic value in patients with intermediate PSA levels (10–20 ng/mL) and advanced age (>75 years). Among younger patients (<50 years) with high PSA (>20 ng/mL) and PI-RADS 5 scores, the detection rate reached 100%, suggesting a potentially high-risk subgroup. Elderly patients (>75 years) consistently exhibited higher detection rates, particularly when PSA exceeded 20 ng/mL or PI-RADS scores were 4–5 (Table 4).

**Table 4:** The sub-analysis of patients with PCa in different age, PSA and PI-RADS levels.

		Age (years)		
PSA (μg/mL)	PI-RADS	<50	50–75	>75
<10	2	0/3 (0.00%)	9/74 (12.16%)	5/13 (38.46%)
	3	1/5 (20.00%)	29/209 (13.88%)	7/24 (29.17%)
	4	0/4 (0.00%)	90/186 (48.39)	15/25 (60.00%)
	5	0/1 (0.00%)	35/47 (74.47%)	14/20 (70.00%)
10–20	2	1/4 (25.00%)	16/89 (17.89%)	4/10 (40.00%)
	3	0/1 (0.00%)	23/170 (13.53%)	11/28 (39.29%)
	4	2/6 (33.33%)	107/185 (57.84%)	36/58 (62.07%)
	5	0/0 (0.00%)	74/83 (89.16%)	26/33 (78.79%)
>20	2	0/0 (0.00%)	7/29 (24.14%)	3/6 (50.00%)
	3	0/1 (0.00%)	19/91 (20.88%)	10/22 (45.45%)
	4	0/0 (0.00%)	87/120 (72.5%)	61/71 (85.92%)
	5	5/5 (100.00%)	326/341 (95.6%)	164/166 (98.80%)

PSA, prostate-specific antigen; PI-RADS, prostate imaging report and data system.

### 3.3 Characteristics and demographics of patients with different Gleason scores

Higher Gleason scores were significantly associated with elevated PSA, PSAD, and PI-RADS scores (*p* < 0.001), whereas age showed no statistically significant difference (*p* > 0.05). For instance, as Gleason scores increased from 6 to 10, mean PSA levels rose from 20.47 ± 21.05 ng/mL to 64.71 ± 35.61 ng/mL, and PI-RADS scores increased from 3.8 ± 0.9 to 4.67 ± 0.62. Age remained comparable across groups (70.27 ± 7.89 to 70.83 ± 8.40 years). These findings indicate that age is not a reliable predictor of Gleason score. Clinically significant PCa (Gleason score >6) accounted for 92.76% of cases, with high-risk disease (Gleason score ≥8) representing 63.85% (Table 5). This suggests a low proportion of early-stage PCa in the studied Chinese cohort, emphasizing the need for expanded screening efforts to facilitate earlier detection and intervention.

**Table 5:** Characteristics of patients with different Gleason scores.

Variate	Gleason scores				
	6	7	8	9	10
Age (mean ± SD)	70.27 ± 7.89	70 ± 8.01	71.2 ± 8.03	71.39 ± 8.4	70.83 ± 8.4
PSA (mean ± SD)	20.47 ± 21.05	24.08 ± 26.51	48.39 ± 36.8	61.46 ± 35.63	64.71 ± 35.61
PV (mean ± SD)	46.03 ± 25.72	39.8 ± 22.26	45.71 ± 35.43	52.08 ± 45.25	55.12 ± 40.08
PSAD (mean ± SD)	0.58 ± 0.72	0.72 ± 1.02	1.34 ± 1.57	1.71 ± 3.4	1.62 ± 1.58
PI-RADS (mean ± SD)	3.8 ± 0.9	4.06 ± 0.85	4.54 ± 0.68	4.68 ± 0.61	4.67 ± 0.62
Number	86	343	383	287	88
Rate (%)	7.25	28.90	32.27	24.18	7.41

PSA, prostate-specific antigen; PSAD, PSA density; PV, prostate volume; PI-RADS, Prostate Imaging Reporting and Data System; PCa, prostate cancer; SD, standard deviation.

### 3.4 Perioperative complications of biopsy

Among 1,078 TRB patients, post-procedural complications included: (1) hematuria (8.4%, *n* = 90): The majority resolved spontaneously within 3 days with increased fluid intake, while 8 patients (0.7%) required hemostatic agents and bladder irrigation. (2) Fever (5.3%, *n* = 57): Five cases (0.5%) progressed to septic shock but recovered after intravenous antibiotics; the remainder were managed successfully with oral antibiotics. (3) Transient urinary retention (5.2%, *n* = 56): All cases resolved with short-term catheterization. In contrast, among 2,130 TPB patients, complications were significantly lower: (1) hematuria (1.22%, *n* = 26): All cases resolved spontaneously within 3 days with conservative management. (2) Fever (2.63%, *n* = 56): All patients responded to oral antibiotics. (3) Transient urinary retention (1.97%, *n* = 42): Required only short-term catheterization. No severe complications (e.g., massive hemorrhage, prostatic abscess, or sepsis) occurred in TPB group (Table 6). Retrospective application of the Clavien-Dindo classification confirmed that all complications occurring during the postoperative hospitalization (median 3 days) were Grade II or lower. Specifically, febrile episodes were diagnosed as urinary tract infections (Grade II) and resolved with sensitive antibiotic therapy. Hematuria and urinary retention were primarily Grade I (managed conservatively) or Grade II (requiring pharmacologic intervention or prolonged catheterization).

**Table 6:** Comparison of safety and efficacy of our study with the reported researches.

Method		TRB				TPB			
Studies		Study I (14)	Study II (15)	Study III (16)	Our study	Study II (15)	Study IV (13)	Study V (12)	Our study
Detection rate (%)		36%	31.9%	29.1%	50.5%	35.3%	45%	43%	55.73%
Complications	Hematuria	1.14%	23.0%	66.3%	8.4%	19.8%	5.3%	NA	1.22%
	Difficulty urinating	9.76%	NA	4.4%	5.2%	NA	3%	5–11.1%	1.97%
	Infection (fever)	6.59%	4.3%	4.7%	5.3%	1.2%	2.2%	2.7%	2.63%

TRB, transrectal prostate biopsy; TPB, transperineal prostate biopsy.

### 3.5 Comparison with other studies in this field

Our study achieved a PCa detection rate of 55.7% using TPB, significantly higher than rates reported in other TPB studies (range: 35.3–43%). Importantly, our complication rates were substantially lower: hematuria (1.2% vs. literature range 10.3–19.8%), urinary retention (2.0% vs. up to 11.1%), and infection (2.6% vs. up to 2.7%). Similarly, our TRB approach yielded a 50.5% detection rate, exceeding reported TRB rates (range: 31.9–37.1%) while maintaining superior safety outcomes: hematuria (8.4% vs. up to 66.3%) and urinary retention (5.2% vs. up to 9.8%). These results demonstrate that the 12 + X-core biopsy strategy provides consistently higher detection rates and lower complication rates regardless of access route (TPB or TRB), establishing its clinical superiority (15–19). The data presented in Tables 2–5 correspond to patients who underwent transperineal prostate biopsy. For a corresponding analysis of the transrectal prostate biopsy data, readers are directed to our previously published work (13, 14).

## 4 Discussion

The PCa exhibits marked geographical disparities in incidence, representing the most prevalent male malignancy in Europe and the United States, whereas its incidence remains relatively lower in Asian populations. However, recent epidemiological data indicate a rising trend in PCa incidence in China (20). Early diagnosis is critical for improving patient prognosis, and prostate biopsy remains the gold standard for definitive diagnosis. Since Hodge et al. first introduced ultrasound-guided prostate biopsy in 1989 (21), two primary approaches have been established: TPB and TRB. Despite multiple randomized controlled trials comparing these techniques, the optimal approach for maximizing detection rates remains controversial (22–24).

To further evaluate the advantages of TPB, we analyzed 2,130 patients who underwent prostate biopsy at Anhui Medical University, focusing on positive detection rates and postoperative complications. Our results demonstrated that the detection rate of TPB 12 + X-core biopsy (55.73%) significantly exceeded those reported for TRB in Taiwan (36%) and Canada (44.6%) (17, 25). Notably, this rate was also higher than the historical TRB detection rate (51%) at our institution from 2014 to 2018 (14), supporting the superiority of TPB in improving diagnostic yield. Consistent with prior evidence, we observed that increased core numbers (e.g., 12 cores vs. 6 sextant biopsies) correlated with higher detection rates (40.4% vs. 32.5%) (26), likely due to enhanced sampling of the prostate volume and reduced risk of missed diagnoses.

The safety of prostate biopsy is a key consideration in clinical practice. In our study, postoperative complications, including hematuria (1.22%), fever (2.63%), and urinary retention (1.97%), were minimal, with no cases of major bleeding or sepsis. These rates were substantially lower than those reported in Taiwan (urinary retention: 9.76%, major bleeding: 1.14% and infection: 6.59%) and Ontario, Canada, where infection-related hospitalizations continue to rise (17, 25). We attribute this favorable safety profile to the TPB approach, which offers both diagnostic efficacy and reduced morbidity.

Age and PSA levels are well-established predictors of PCa, with advanced age constituting a significant risk factor (27, 28). Current guidelines emphasize PCa screening in elderly males to facilitate early diagnosis and treatment (12, 28). Our findings corroborated a positive association between age and PCa detection, with older patients (particularly those >75 years) exhibiting elevated malignancy risk even at lower PSA levels or PI-RADS scores. While high-risk PCa cases (based on biopsy) predominantly occurred in patients aged >70, age did not further stratify risk within this subgroup, suggesting its role in tumor initiation rather than progression. PSA, though widely used for screening, suffers from limited specificity due to confounding factors such as benign prostatic hyperplasia, prostatitis, or instrumentation (29). Combining PSA with other markers (e.g., testosterone, alkaline phosphatase) may improve diagnostic accuracy (30). Additionally, mp-MRI with PI-RADS scoring significantly enhanced diagnostic sensitivity and specificity in our cohort, aligning with prior research (31).

To assess the clinical utility of 12 + X-core TPB, we evaluated age, PSA, PSAD, and PI-RADS scores as predictors. PCa patients exhibited higher mean values for all parameters compared to BPH patients, alongside smaller prostate volumes, a finding potentially explained by compression of the peripheral zone in larger prostates (24). Elevated PSA (>20 ng/mL) and PI-RADS scores (≥4) were strongly associated with PCa detection, particularly in older males. Moreover, rising Gleason scores correlated with incremental increases in PSA, PSAD, and PI-RADS, reflecting tumor aggressiveness and supporting earlier observations (27).

The definition of “high-risk” PCa in this study (Gleason score ≥8) deviates from some conventional systems that include Gleason 7 disease. While this choice was intentional to sharpen our focus on the most aggressive tumor phenotypes, it inevitably increases the reported proportion of “high-risk” cancers and may limit direct comparability with studies employing broader definitions. Future analyses would benefit from presenting outcomes across all standard risk groups.

Our study, which utilized a combined approach of targeted and systematic biopsy, naturally engages with the contemporary debate regarding the potential omission of systematic cores in patients with a visible MRI lesion. While targeted biopsy alone is known to improve the detection of clinically significant cancer, a growing body of evidence suggests that relying solely on targeted cores may miss a non-negligible proportion of significant tumors. This is particularly true for tumors that are MRI-invisible or located in regions outside the index lesion (32). Our findings, which demonstrated the value of the combined approach, align with studies that caution against abandoning systematic biopsy prematurely, especially in biopsy-naïve patients or those with heterogeneous or large prostates. However, we also acknowledge the compelling argument for omitting systematic cores in specific scenarios, such as repeat biopsies or when using advanced fusion platforms with high accuracy, to reduce morbidity and procedure time. Future research should focus on better identifying patient subgroups in whom a biopsy strategy omitting systematic cores can be safely adopted, potentially guided by more precise imaging or molecular tools (10, 33). The accuracy of our biopsy strategy is further reflected in the concordance of Gleason scores between biopsy and radical prostatectomy specimens, a critical metric for guiding appropriate management. Discrepancies, particularly upgrades from biopsy to surgery, can significantly alter prognosis and treatment recommendations. Our observed concordance rate of 82.6% is situated within the spectrum reported in recent literature (34). These variations can be attributed to several factors, including inherent tumor heterogeneity, the sampling limitation of biopsy, and inter-observer variability in pathological interpretation. The integration of more extensive targeted sampling, as performed in our protocol, has been shown to improve this concordance by better capturing the index lesion’s highest grade. Nonetheless, the persistent risk of upgrading underscores the limitation of biopsy as a sampling procedure and highlights the potential future role of predictive tools, such as artificial intelligence-based models that analyze biopsy core textures or MRI features, to pre-operatively identify tumors at high risk of being upgraded.”

Beyond histological architecture, the field is increasingly moving towards a molecular understanding of PCa aggressiveness (11, 35–37). There is a well-documented correlation between higher Gleason Grade Groups (GGG) and specific molecular alterations, such as PTEN loss, TP53 mutations, and other genomic aberrations associated with more aggressive disease. While the studies focused on the histopathological grade, the findings on the distribution of GGG in our cohort provide a morphological backdrop for this molecular continuum. The tumors identified as GGG 4 or 5, for instance are likely to harbor a higher burden of these adverse molecular markers (38). Future paradigms for PCa diagnosis and risk stratification will undoubtedly involve the integration of histopathological findings from biopsies with molecular subtyping. The development of foundation models and AI algorithms that can correlate imaging phenotypes with both histological grade and molecular signatures represents a promising frontier. Positioning our biopsy strategy within this future framework suggests its potential not only for accurate grading but also as a source of tissue for subsequent molecular profiling, ultimately enabling more personalized treatment decisions.”

Despite demonstrating the clinical utility of 12 + X-core transperineal ultrasound-guided prostate biopsy through a large cohort analysis, this study has several limitations: (1) Single-center design: The data were derived from a single institution in a specific geographical region, and the study population was restricted to a defined time period. Consequently, the findings may be subject to selection bias and lack generalizability across diverse populations or practice settings. (2) Retrospective study: As a retrospective analysis, the study is inherently susceptible to biases in data collection and patient selection, which may influence the interpretation of outcomes. (3) Temporal bias between cohorts: The comparison between the TPB (2018–2024) and TRB (2015–2018) groups introduces a substantial risk of time-related confounding. Factors such as improvements in mp-MRI quality, increased operator proficiency in cognitive fusion, and shifts in patient demographics or referral patterns over time may have independently contributed to the higher detection rates and improved safety outcomes observed in the TPB cohort. Although we adjusted for known variables in multivariate analyses, unmeasured confounding may remain.

## 5 Conclusion

Ultrasound-guided TPB with a 12 + X-core scheme combined with MRI-cognitive fusion demonstrates superior clinical utility by enabling precise lesion targeting. This approach significantly improves the detection of clinically significant and high-risk PCa while minimizing procedure-related complications compared to TRB method. Consequently, for men with clinical suspicion of PCa requiring biopsy, the TPB with a 12 + X-core protocol might represent a robust and preferable diagnostic method.

## Data Availability

The original contributions presented in the study are included in the article/Supplementary material, further inquiries can be directed to the corresponding authors.

## References

[1] Siegel RL Giaquinto AN Jemal A. Cancer statistics, 2024. CA Cancer J Clin. (2024) 74:12–49. doi: 10.3322/caac.21820, PMID: 38230766

[2] Raychaudhuri R Lin DW Montgomery RB. Prostate cancer: a review. JAMA. (2025) 333:1433–46. doi: 10.1001/jama.2025.0228, PMID: 40063046

[3] Smelik M Diaz-Roncero GD An X Heer R Henningsohn L Li X . Combining spatial transcriptomics, pseudotime, and machine learning enables discovery of biomarkers for prostate cancer. Cancer Res. (2025) 85:2514–26. doi: 10.1158/0008-5472.CAN-25-0269 40293712 PMC12214874

[4] Pakzad R Mohammadian-Hafshejani A Ghoncheh M Pakzad I Salehiniya H. The incidence and mortality of prostate cancer and its relationship with development in Asia. Prostate Int. (2015) 3:135–40. doi: 10.1016/j.prnil.2015.09.001, PMID: 26779461 PMC4685206

[5] Litwin MS Tan HJ. The diagnosis and treatment of prostate cancer: a review. JAMA. (2017) 317:2532–42. doi: 10.1001/jama.2017.7248, PMID: 28655021

[6] Xiang J Yan H Li J Wang X Chen H Zheng X. Transperineal versus transrectal prostate biopsy in the diagnosis of prostate cancer: a systematic review and meta-analysis. World J Surg Oncol. (2019) 17:31. doi: 10.1186/s12957-019-1573-0, PMID: 30760274 PMC6375152

[7] Grummet JP Weerakoon M Huang S Lawrentschuk N Frydenberg M Moon DA . Sepsis and ‘superbugs’: should we favour the transperineal over the transrectal approach for prostate biopsy? BJU Int. (2014) 114:384–8. doi: 10.1111/bju.12536, PMID: 24612341

[8] Becerra MF Alameddine M Zucker I Tamariz L Palacio A Nemeth Z . Performance of multiparametric MRI of the prostate in biopsy naive men: a meta-analysis of prospective studies. Urology. (2020) 146:189–95. doi: 10.1016/j.urology.2020.06.102, PMID: 32890616

[9] Wang R Wang J Gao G Hu J Jiang Y Zhao Z . Prebiopsy mp-MRI can help to improve the predictive performance in prostate cancer: a prospective study in 1,478 consecutive patients. Clin Cancer Res. (2017) 23:3692–9. doi: 10.1158/1078-0432.CCR-16-2884, PMID: 28143868

[10] Pepe P Pepe L Fiorentino V Curduman M Pennisi M Fraggetta F. PSMA PET/CT accuracy in diagnosing prostate cancer nodes metastases. In Vivo. (2024) 38:2880–5. doi: 10.21873/invivo.13769, PMID: 39477430 PMC11535925

[11] Fiorentino V Pepe L Pizzimenti C Zuccalà V Pepe P Cianci V . PD-L1 expression in prostate cancer and Gleason grade group: is there any relationship? Findings from a multi-institutional cohort. Pathol Res Pract. (2025) 269:155916. doi: 10.1016/j.prp.2025.155916, PMID: 40107012

[12] Cornford P van den Bergh RCN Briers E van den Broeck T Brunckhorst O Darraugh J . EAU-EANM-ESTRO-ESUR-ISUP-SIOG guidelines on prostate cancer-2024 update. Part I: screening, diagnosis, and local treatment with curative intent. Eur Urol. (2024) 86:148–63. doi: 10.1016/j.eururo.2024.03.027, PMID: 38614820

[13] Wang H Tai S Zhang L Zhou J Liang C. A calculator based on Prostate Imaging Reporting and Data System version 2 (PI-RADS V2) is a promising prostate cancer predictor. Sci Rep. (2019) 9:6870. doi: 10.1038/s41598-019-43427-9, PMID: 31053749 PMC6499813

[14] Wang H Tai S Zhang L Zhou J Liang C. A new predictor is comparable to the updated nomogram in predicting the intermediate- and high-risk prostate cancer but outperforms nomogram in reducing the overtreatment for the low-risk Pca. Cancer Manag Res. (2019) 11:3753–63. doi: 10.2147/CMAR.S194258, PMID: 31118794 PMC6500873

[15] Mian BM Feustel PJ Aziz A Kaufman RP Jr Bernstein A Avulova S . Complications following transrectal and transperineal prostate biopsy: results of the ProBE-PC randomized clinical trial. J Urol. (2024) 211:205–13. doi: 10.1097/JU.0000000000003788, PMID: 37976319

[16] Huang GL Kang CH Lee WC Chiang PH. Comparisons of cancer detection rate and complications between transrectal and transperineal prostate biopsy approaches - a single center preliminary study. BMC Urol. (2019) 19:101. doi: 10.1186/s12894-019-0539-4, PMID: 31660936 PMC6816188

[17] Wei TC Lin TP Chang YH Chen TJ Lin AT Chen KK. Transrectal ultrasound-guided prostate biopsy in Taiwan: a nationwide database study. J Chin Med Assoc. (2015) 78:662–5. doi: 10.1016/j.jcma.2015.04.011, PMID: 26239148

[18] Guo LH Wu R Xu HX Xu JM Wu J Wang S . Comparison between ultrasound guided transperineal and transrectal prostate biopsy: a prospective, randomized, and controlled trial. Sci Rep. (2015) 5:16089. doi: 10.1038/srep16089, PMID: 26526558 PMC4630643

[19] Efesoy O Bozlu M Cayan S Akbay E. Complications of transrectal ultrasound-guided 12-core prostate biopsy: a single center experience with 2,049 patients. Turk J Urol. (2013) 39:6–11. doi: 10.5152/tud.2013.002, PMID: 26328070 PMC4548577

[20] Bray F Laversanne M Sung H Ferlay J Siegel RL Soerjomataram I . Global cancer statistics 2022: GLOBOCAN estimates of incidence and mortality worldwide for 36 cancers in 185 countries. CA Cancer J Clin. (2024) 74:229–63. doi: 10.3322/caac.21834, PMID: 38572751

[21] Hodge KK McNeal JE Terris MK Stamey TA. Random systematic versus directed ultrasound guided transrectal core biopsies of the prostate. J Urol. (1989) 142:71–4. doi: 10.1016/s0022-5347(17)38664-0, PMID: 2659827

[22] Pepe P Garufi A Priolo G Pennisi M. Transperineal versus transrectal MRI/TRUS fusion targeted biopsy: detection rate of clinically significant prostate cancer. Clin Genitourin Cancer. (2017) 15:e33–6. doi: 10.1016/j.clgc.2016.07.007, PMID: 27530436

[23] Cerruto MA Vianello F D'Elia C Artibani W Novella G. Transrectal versus transperineal 14-core prostate biopsy in detection of prostate cancer: a comparative evaluation at the same institution. Arch Ital Urol Androl. (2014) 86:284–7. doi: 10.4081/aiua.2014.4.284, PMID: 25641452

[24] Abdollah F Novara G Briganti A Scattoni V Raber M Roscigno M . Trans-rectal versus trans-perineal saturation rebiopsy of the prostate: is there a difference in cancer detection rate? Urology. (2011) 77:921–5. doi: 10.1016/j.urology.2010.08.048, PMID: 21131034

[25] Nam RK Saskin R Lee Y Liu Y Law C Klotz LH . Increasing hospital admission rates for urological complications after transrectal ultrasound guided prostate biopsy. J Urol. (2010) 183:963–9. doi: 10.1016/j.juro.2009.11.043, PMID: 20089283

[26] Ploussard G Nicolaiew N Marchand C Terry S Vacherot F Vordos D . Prospective evaluation of an extended 21-core biopsy scheme as initial prostate cancer diagnostic strategy. Eur Urol. (2014) 65:154–61. doi: 10.1016/j.eururo.2012.05.049, PMID: 22698576

[27] Pepe P Aragona F. Morbidity after transperineal prostate biopsy in 3,000 patients undergoing 12 vs. 18 vs. more than 24 needle cores. Urology. (2013) 81:1142–6. doi: 10.1016/j.urology.2013.02.019, PMID: 23726443

[28] Freedland SJ Friedrich NA. Nature versus nurture contribution to prostate cancer risk. Nat Rev Urol. (2022) 19:635–6. doi: 10.1038/s41585-022-00650-w, PMID: 36042289 PMC10179443

[29] Guzman JA Sharma P Smith LA Buie JD de Riese WT. Histological changes of the peripheral zone in small and large prostates and possible clinical implications. Res Rep Urol. (2019) 11:77–81. doi: 10.2147/RRU.S182781 30963056 PMC6432882

[30] Stephan C Rittenhouse H Hu X Cammann H Jung K. Prostate-specific antigen (PSA) screening and new biomarkers for prostate cancer (PCa). EJIFCC. (2014) 25:55–78. Available at: https://pmc.ncbi.nlm.nih.gov/articles/PMC4975191/ PMID: 27683457 PMC4975191

[31] Hammerich KH Donahue TF Rosner IL Cullen J Kuo HC Hurwitz L . Alkaline phosphatase velocity predicts overall survival and bone metastasis in patients with castration-resistant prostate cancer. Urol Oncol. (2017) 35:460.e21–8. doi: 10.1016/j.urolonc.2017.02.001, PMID: 28410987

[32] Barentsz JO Weinreb JC Verma S Thoeny HC Tempany CM Shtern F . Synopsis of the PI-RADS v2 guidelines for multiparametric prostate magnetic resonance imaging and recommendations for use. Eur Urol. (2016) 69:41–9. doi: 10.1016/j.eururo.2015.08.038, PMID: 26361169 PMC6364687

[33] Pepe P Pepe L Fiorentino V Curduman M Fraggetta F. Multiparametric MRI targeted prostate biopsy: when omit systematic biopsy? Arch Ital Urol Androl. (2024) 96:12992. doi: 10.4081/aiua.2024.12992, PMID: 39692414

[34] Pepe P Pepe L Tamburo M Marletta G Savoca F Pennisi M . 68Ga-PSMA PET/CT evaluation in men enrolled in prostate cancer active surveillance. Arch Ital Urol Androl. (2023) 95:11322. doi: 10.4081/aiua.2023.11322 37212907

[35] Fiorentino V Martini M Dell’Aquila M Musarra T Orticelli E Larocca LM . Histopathological ratios to predict Gleason score agreement between biopsy and radical prostatectomy. Diagnostics. (2020) 11:10. doi: 10.3390/diagnostics11010010 33374618 PMC7822416

[36] Kiełb P Kowalczyk K Gurwin A Nowak Ł Krajewski W Sosnowski R . Novel histopathological biomarkers in prostate cancer: implications and perspectives. Biomedicine. (2023) 11:1552. doi: 10.3390/biomedicines11061552, PMID: 37371647 PMC10295349

[37] Vlajnic T Bubendorf L. Molecular pathology of prostate cancer: a practical approach. Pathology. (2021) 53:36–43. doi: 10.1016/j.pathol.2020.10.003, PMID: 33234230

[38] Pecci V Troisi F Aiello A De Martino S Carlino A Fiorentino V . Targeting of H19/cell adhesion molecules circuitry by GSK-J4 epidrug inhibits metastatic progression in prostate cancer. Cancer Cell Int. (2024) 24:56. doi: 10.1186/s12935-024-03231-6, PMID: 38317193 PMC10845766

