# Supplementary material for: Comparative analysis of ultrasound-guided magnetic resonance imaging-cognitive fusion transrectal versus transperineal prostate biopsy: a 10-year single-center retrospective analysis
Source: Front Med (Lausanne). 2025 Nov 19;12:1713863. doi: 10.3389/fmed.2025.1713863 (PMC12672434; doi:10.3389/fmed.2025.1713863)
Supplement: Supplementary file 1 [file Supplementary_file_1.docx]

**Supplementary file**

**Comparative Analysis of Ultrasound-Guided Magnetic Resonance Imaging-Cognitive Fusion Transrectal versus Transperineal Prostate Biopsy: A 10-year Single-center Retrospective Analysis**

***Jincheng Luo^#1^, Jie Sun^#1,2^, Hui Wang^*1^, Cheng Yang^*1^***

1. Department of Urology, The First Affiliated Hospital of Anhui Medical University, Institute of Urology, Anhui Medical University, and Anhui Province Key Laboratory of Urological and Andrological Diseases Research and Medical Transformation, Hefei 230022, China.
2. Department of Urology, The Fourth People’s Hospital of Lu’an, Lu’an 237005, China.

^#^ These authors made an equal contribution to the research.

^*^Corresponding author:

Hui Wang, Department of Urology, The First Affiliated Hospital of Anhui Medical University, No. 218 Jixi Road, Hefei, 230022, China. Email: [whayd@sina.cn](mailto:whayd@sina.cn).

Cheng Yang, Department of Urology, The First Affiliated Hospital of Anhui Medical University, No. 218 Jixi Road, Hefei, 230022, China. Email: yang_cheng@ahmu.edu.cn.
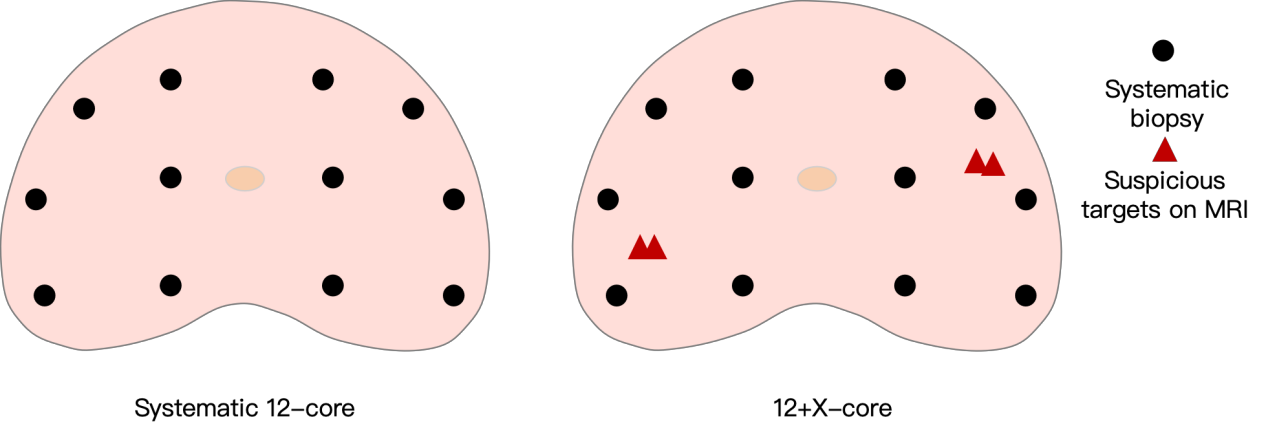


**Supplementary Figure 1**. The distribution of prostate biopsy cores.
